# Autologous conditioned serum for implant site enhancement

**DOI:** 10.34172/japid.2022.005

**Published:** 2022-04-12

**Authors:** Zeinab Torab, Hamidreza Mohammadi

**Affiliations:** ^1^Department of Pediatric Dentistry, Faculty of Dentistry, Tabriz University of Medical Sciences, Tabriz, Iran; ^2^Department of Periodontics, Faculty of Dentistry, Tabriz University of Medical Sciences, Tabriz, Iran

 Many traumatic events lead to bone loss in different areas of the dental ridge. These traumatic events include tooth loss, sinus pneumatization, periodontal disease, etc.^1^ Bone deficiency in different areas is problematic in terms of implant placement, necessitating horizontal bone augmentation and socket preservation methods to improve hard tissue conditions in the area.^2^Changes in histological, histomorphometric, and radiographic properties of hard tissue in the area of implant placement affect many parameters of implant success.^3^ Improving characteristics such as alkaline phosphatase enzyme activity and the rate at which new living bone is formed and its pattern of mineralization and trabeculae are essential for achieving a high success rate for implants placed in the area.^3^ Many destructive processes in dental ridge areas, in addition to microbial etiology, involve the response of host cells through inflammatory mediators.

 As a potent inflammatory mediator, IL-1 is involved in many of the body’s inflammatory processes and is responsible for many restorative outcomes of therapeutic interventions in the implant area.^4^This mediator is released in physiological amounts from different cells such as macrophages in the area and affects the progression of inflammation and ultimate tissue repair.^5^ However, in high amounts, it causes more inflammation and destructive processes, including bone loss.^4^ It seems necessary to control and modulate the host’s inflammatory response in situ to affect the secretion of various cytokines and activate or inhibit their activity, depending on the time and place of intervention, reduce the destructive processes, and improve tissue properties. Autologous conditioned serum (ACS) is a blood product that has a high level of IL-1 receptor antagonists. The IL-1 receptor antagonist is also found naturally in the body. ACS was first used as a new therapeutic agent in the mid-1990s to treat osteoarthritis in injectable form with high levels of the IL-1 receptor antagonist. This product is used topically to treat and improve bone resorption and inflammation of the area where IL-1 is the main cause. ACS is used to treat degenerative joint diseases, especially knee osteoarthritis.^5^ Today, prefabricated forms of this product are also available, which has become very popular for treating degenerative joint events.^6^It seems that the use of this product is useful and necessary for periodontal regenerative processes and implantation site enhancement. In a study, injecting an IL-1β antagonist to treat arthritis in rats and humans inhibited the IL-1Ra receptor, preventing the destruction of the extracellular matrix of chondrocytes.^7^ One study examined the effect of commercially available ACS on temporomandibular joint inflammatory disorders and reported promising results in treating TMJ arthritis using this product.^8^Another study reported that injecting commercially available ACS to treat arthritis decreased joint pain and improved joint function. They attributed this improvement in joint function to decreased inflammation and reduced joint damage.^9^

 Augmentation surgeries are required in many cases because implant placement in the edentulous areas requires a sufficient amount of bone, and the bone in many edentulous ridges is dimensionally problematic or will be problematic in the future.

 It seems the use of this material would be useful in procedures to regenerate bone defects and enhance histologic characteristics. To achieve the mentioned goals, it is suggested that more studies be carried out in this field.

## Authors’ contributions

 ZT conceptualized the idea, and HM wrote the letter. Both authors read and approved the final submission.

## Competing interests

 The authors declare that they have no competing interests concerning the authorship and/or publication of this article.
